# LncRNA XIST/miR-34a axis modulates the cell proliferation and tumor growth of thyroid cancer through MET-PI3K-AKT signaling

**DOI:** 10.1186/s13046-018-0950-9

**Published:** 2018-11-21

**Authors:** Hua Liu, Haoyu Deng, Yajie Zhao, Can Li, Yu Liang

**Affiliations:** 1 Nuclear Medicine Department, Xiangya Hospital, Central South University, Changsha, Hunan People’s Republic of China; 2 Oncology Department, Xiangya Hospital, Central South University, Changsha, No. 87 Xiangya Road, Changsha, Hunan 410008 People’s Republic of China

**Keywords:** Thyroid cancer, XIST, miR-34a, MET, PI3K/AKT

## Abstract

**Background:**

Thyroid cancer is one of the most prevalent malignancies in endocrine system. Further understanding and revealing the molecular mechanism underlying thyroid cancer are indispensable for the development of effective diagnosis and treatments. In the present study, we attempted to provide novel basis for targeted therapy for thyroid cancer from the aspect of lncRNA-miRNA-mRNA interaction.

**Methods:**

The expression and cellular function of XIST (X-inactive specific transcript) was determined. miRNAs which may be direct targets of XIST were screened for from online GEO database and miR-34a was selected. Next, the predicted binding between XIST and miR-34a, and the dynamic effect of XIST and miR-34a on downstream MET (hepatocyte growth factor receptor)-PI3K (phosphoinositide 3-kinase)-AKT (α-serine/threonine-protein kinase) signaling was evaluated.

**Results:**

XIST was significantly up-regulated in thyroid cancer tissues and cell lines; XIST knockdown suppressed the cell proliferation in vivo and the tumor growth in vitro. Based on online database and online tool prediction results, miR-34a was underexpressed in thyroid cancer and might be a direct target of XIST. Herein, we confirmed the negative interaction between XIST and miR-34a; moreover, XIST knockdown could reduce the protein levels of MET, a downstream target of miR-34a, and the phosphorylation of PI3K and AKT. In thyroid cancer tissues, MET mRNA and protein levels of MET were up-regulated; MET was positively correlated with XIST while negatively correlated with miR-34a, further confirming that XIST serves as a ceRNA for miR-34a through sponging miR-34a, competing with MET for miR-34a binding, and finally modulating thyroid cancer cell proliferation and tumor growth.

**Conclusion:**

In the present study, we provided novel experimental basis for targeted therapy for thyroid cancer from the aspect of lncRNA-miRNA-mRNA interaction.

**Electronic supplementary material:**

The online version of this article (10.1186/s13046-018-0950-9) contains supplementary material, which is available to authorized users.

## Introduction

Thyroid cancer is a prevalent malignancy in endocrine system; during the past decades, the morbidity and mortality of thyroid cancer is persistently increasing [1]. Thyroid cancers develop from two different cell types: follicular cells and parafollicular (C) cells. Over 90% of thyroid cancer arises from follicular cells, a kind of epithelial cells acting on iodine uptake and thyroid hormone synthesis [2]. Thyroid cancers of follicular origin are the most commonly diagnosed and has been sub-classified as follicular (FTC), papillary (PTC), partially differentiated (PDTC) or anaplastic (ATC) thyroid cancer. The FTC and PTC forms are characterized as differentiated cancers, and the PTCs alone account for approximately 80% of all thyroid tissue malignancies [3, 4]. However, the pathogenesis of thyroid cancers of different pathological patterns is distinct from each other [5]. Investigating the underlying mechanism of the carcinogenesis of thyroid cancer is indispensable for developing more effective strategies of diagnosis and therapy.

The role of non-coding RNAs in carcinogenesis has been widely reported during the past decades. Long non-coding RNAs (lncRNAs), a series of non-coding RNAs with a length of > 200 nucleotides, have been regarded as crucial regulators of many cancers playing a tumor suppressive or oncogenic role, depending on circumstances. LncRNAs, such as MALAT1, HOTAIR, TUG1, XIST and so on, could promote cancer cell proliferation, invasion and/or migration, including multiple myeloma [6], triple-negative breast cancer [7], cervical cancer [8, 9], pancreatic cancer [10], bladder cancer [11], as well as thyroid cancer [12–15]. Interestingly, lncRNAs exert their biological effects on cancers through interacting with miRNAs, another series of non-coding RNAs containing about 22 nucleotides, functioning as competing endogenous RNAs (ceRNAs) [16, 17].

Herein, we attempted to identify lncRNA-miRNA pair which might interact with each other thus modulating the cell and tumor growth of thyroid cancer based on data from The Cancer Genome Atlas Thyroid Cancer (TCGA-THCA) data collection combined with the prediction results of online tool. Among a large amount of dysregulated lncRNAs, XIST expression was significantly increased in thyroid cancer tissues (Additional file 1: Table S1); moreover, XIST has been regarded as an oncogene in many cancers through interacting with different miRNAs, such as miR-152 [18], miR-101 [19], miR-124 [20], miR-29c [21], miR-29a [22], miR-140 [23], miR-367/141 [24], and miR-133a [25]. However, whether XIST plays a potential role in thyroid cancer through interacting with miRNA remains unclear.

Herein, we used online tool lnc Tar (http://www.cuilab.cn/lnctar) [26] to search for downstream target miRNAs of XIST, and further investigated the function and molecular mechanism by which XIST-miRNA modulated thyroid cancer cell and tumor growth. Furthermore, the possible downstream signaling was also verified. Taken together, we provided novel experimental basis for targeted therapy for thyroid cancer from the aspect of lncRNA-miRNA-mRNA interaction.

## Materials and methods

### Tissue samples, cell lines and cell transfection

A total of 77 paired thyroid cancer and non-tumor tissue samples were collected from patients who underwent surgical resection Xiangya Hospital, Central South University (Changsha, China). All the tissue samples were snap-frozen and stored at − 80 °C.

A human follicular thyroid carcinoma cell line, FTC133 derived from a lymph node metastasis of a follicular thyroid carcinoma, a human thyroid cancer papillary cell line, BCPAP, and a normal human thyroid epithelium cell line (TEC) were purchased from the ScienCell Research Laboratories (Carlsbad, CA, USA). A human papilloma thyroid carcinoma cell line, TPC-1 cell line was purchased from the American Type Culture Collection (ATCC, Manassas, VA, USA). Human thyroid cell lines SW1736 and KAT18 were purchased from the Tumor Cell Bank of the Chinese Academy of Medical Science (Shanghai, China). The above cells were cultured in RPMI-1640 medium (Sigma, St. Louis, MO, USA) supplemented with 10% fetal bovine serum (Life Technologies Corp., USA), streptomycin (100 U/ml) and penicillin sodium (100 U/ml) at 37 °C in a humidified atmosphere with 5% CO_2_.

XIST expression was achieved by transfection of si-XIST#1 or si-XIST#2 (GeneCopoecia, Guangzhou, China). The overexpression of miRNAs was achieved by transfection of specific miRNA mimics (Genepharma, Shanghai, China) with the help of Lipofectamine 2000 (Invitrogen). MiR-34a inhibition was achieved by transfection of miR-34a inhibitor (Genepharma, Shanghai, China).

### Real-time PCR

Total RNA was extracted using Trizol reagent (Invitrogen) following the protocol. By using miRNA-specific primer, total RNA was reverse transcribed and the miScript Reverse Transcription kit (Qiagen, Germany) was used for miRNA qRT-PCR. The RNU6B expression was used as an endogenous control. The SYBR green PCR Master Mix (Qiagen) was used for mRNA expression detection following the protocol. The GAPDH expression was used as an endogenous control. 2^−ΔΔ^CT method was used to analyze the relative fold changes. All sequences used are provided in Additional file 1: Table S2.

### MTT assay

A modified MTT assay was used to evaluate cell viability. 24 h after seeding into 96-well plates (5 × 10^3^ cells/well), cells were transfected and/or treated as described. 48 h after transfection, 20 μl MTT (at a concentration of 5 mg/ml; Sigma-Aldrich) was added, and the cells were incubated for an additional 4 h in a humidified incubator. 200 μl DMSO was added after the supernatant discarded to dissolve the formazan. OD_490 nm_ value was measured. The viability of the non-treated cells (control) was defined as 100%, and the viability of cells from all other groups was calculated separately from that of the control group.

### BrdU incorporation assay

DNA synthesis in proliferating cells was determined by measuring 5-Bromo-2-deoxyUridine (BrdU) incorporation. BrdU assays were performed at 24 h and 48 h after infection. After seeding the infected cells in 96-well culture plates at a density of 2 × 10^3^ cells/well, they were cultured for 24 h or 48 h, and incubated with a final concentration of 10 μM BrdU (BD Pharmingen, San Diego, CA, USA) for 2 h to 24 h. When the incubation period ended, we removed the medium, fixed the cells for 30 min at RT, incubated them with peroxidase-coupled anti-BrdU-antibody (Sigma-Aldrich) for 60 min at RT, washed them three times with PBS, incubated the cells with peroxidase substrate (tetramethylbenzidine) for 30 min, and measured the absorbance values at 450 nm. Background BrdU immunofluorescence was determined in cells not exposed to BrdU but stained with the BrdU antibody.

### Xenograft tumor model

A total of 30 eight-week-old athymic female nude mice with an average weight of 20 g were obtained from The Jackson Laboratory (Bar Harbor, ME USA), and anesthetized with an intraperitoneal injection of 2% 2,2,2-Tribromoethanol (200 μl/mouse; Sigma) before implantation of KAT18 or TPC-1 thyroid cancer cells (si-NC, si-XIST#1 or si-XIST#2 transfected). Flank tumors were established by injecting 1 × 10^6^ cells in 100 μL of ECM gel (Sigma) into the subcutaneous flanks of nude mice. Tumor dimensions were serially measured with electronic calipers, and the volumes were calculated by the following formula: a (the largest diameter) × b^2^ (the perpendicular diameter) × 0.4. The body weight of each animal was followed as a marker of toxicity. The humane endpoints were a tumor diameter ≥ 2.0 cm, significant weight loss (20% of pre-experiment body weight), weight loss to a final weight of 16 g, very slow breathing rate, shallow or labored breathing pattern, decreased activity, poor response to handling, absence of grooming, social isolation, hunched posture, shivering, and muscle atrophy. The method of euthanasia was CO_2_ exposure for 10 min, at a 20% fill rate of cage volume/min. The animal protocol for this experiment has been maintained in accordance with the guidelines of the animal care committee of Xiangya Hospital, Central South University. All procedures were conducted under sterile conditions. The mice were allowed access to sterile food and water ad libitum.

### Luciferase reporter assay

The fragment of XIST was amplified by PCR and cloned to the downstream of the Renilla psiCHECK2 vector (Promega, Madison, WI, USA), named wt-XIST. To generate the XIST mutant reporter, the seed region of the XIST was mutated to remove all complementarity to nucleotides 2–7 of miR-34a, named mut-XIST. HEK293 cells (ATCC, USA) were seeded into a 24-well plate. After cultured overnight, HEK293 cells were co-transfected with the indicated vectors and miR-34a mimics or miR-34a inhibitor, respectively. Luciferase assays were performed 48 h after transfection using the Dual Luciferase Reporter Assay System (Promega). Renilla luciferase activity was normalized to Firefly luciferase activity for each transfected well. All sequences used are provided in Additional file 1: Table S2.

### RNA immunoprecipitation (RIP)

RNA immunoprecipitation was performed using Magna RIP RNA-Binding Protein Immunoprecipitation Kit (17–700, Millipore) according to manufacturer’s instructions. RNA for in vitro experiments was transcribed using T7 High YieldRNA Synthesis Kit (E2040S, NEB) according to manufacturer’s instructions. IgG, XIST and miR-34a levels in the immunoprecipitates were measured by qRT-PCR.

### Immunoblotting assays

The protein levels of MET, p-PI3K, PI3K, p-AKT and AKT in thyroid cancer cells were detected by performing immunoblotting assays. Cells were lysed by RIPA buffer (Sigma-Aldrich, USA) with Complete Protease Inhibitor Cocktail (Roche, USA). Cell lysates were transferred to 1.5 mL tube and kept at − 20 °C before use. SDS-PAGE was conducted to separate the cellular proteins. Proteins were loaded onto SDS-PAGE minigel, and then transferred onto PVDF membrane. The blots were probed with the following antibodies at 4 °C overnight: anti-MET (ab51067, Abcam, Cambridge, MA, USA), anti-p-PI3K (phospho Y607, ab182651, Abcam), anti-PI3K (ab86714, Abcam), anti-p-AKT (phospho S473, ab81283, Abcam), anti-AKT (ab179463, Abcam) and anti-GAPDH (ab8245, Abcam) and incubated with HRP-conjugated secondary antibody (1:5000). Signals were visualized using ECL Substrates (Millipore, USA). The protein expression was normalized to endogenous GAPDH.

### Statistical analysis

Data are processed using SPSS17.0 statistical software and presented as the mean ± S.D. of results from at least three independent experiments. A Student *t* test was used for statistical comparison between means where applicable. Differences among more than two groups in the above assays were estimated using one-way ANOVA. *P < 0.05; **P < 0.01.

## Results

### The expression of XIST and its correlation with the clinical parameters in patients with thyroid cancer

**Fig. 1:**
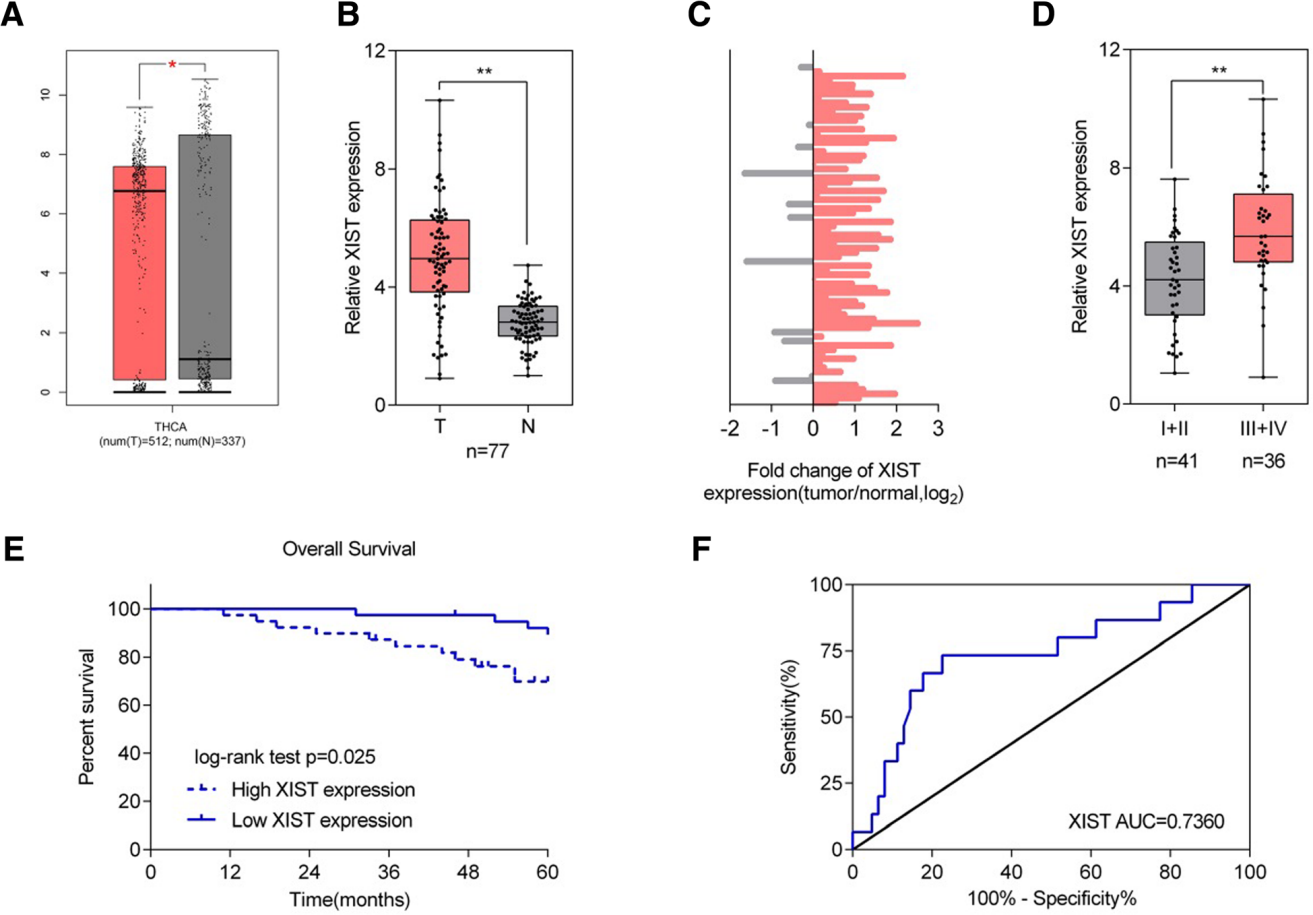
Expression of XIST and its correlation with the clinical parameters in patients with thyroid cancer (**a**) XIST expression in 512 thyroid cancer tissue samples and 337 adjacent non-tumor tissue samples based on the data from TCGA-THCA were exhibited. **b** XIST expression in 77 paired thyroid cancer tissue samples and adjacent non-tumor tissue samples was examined using real-time PCR assays. **c** The distribution of XIST expression fold-change shown as tumor/normal (log_2_). **d** XIST expression in tumor tissues grouping by TNM stages (I + II vs. III + IV). The data are presented as mean ± SD of three independent experiments. *P < 0.05, **P < 0.01. **e** Kaplan-Meier overall survival curves for 77 patients with thyroid cancer classified according to relative XIST expression level. (F) Receiver operating characteristic (ROC) curve showing the area under the curve (AUC) to analyze the sensitivity and specificity of XIST as a biomarker for thyroid cancer

Briefly, the expression of XIST in thyroid cancer and adjacent non-tumor tissues was first examined. As shown in Fig. 1a, XIST expression was significantly increased in thyroid cancer tissue samples, compared to adjacent non-tumor tissue samples, based on the data from TCGA-THCA. In a total of 77 paired thyroid cancer and non-tumor tissue samples, we detected the expression of XIST using real-time PCR assays. Consistent with the above data, XIST expression was remarkably up-regulated in thyroid tissues compared to that in non-tumor tissues (Fig. 1b-c). These 77 cases were divided into two groups according to XIST expression: a high XIST group (XIST expression above the median value, *n* = 39) and a low XIST group (XIST expression below the median value, *n* = 38). The correlation of clinical parameters with XIST expression was analyzed and shown in Table 1. As shown in Fig. 1d, XIST expression was higher in tissue samples derived from patients in advanced stages (III + IV), compared to that in earlier stages (I + II). Moreover, higher XIST expression was correlated with larger tumor size, advanced N stages and TNM stages (Table 1). A COX risk proportional regression model was used to analyze the overall survival time and the clinical parameters of 77 patients. Univariate analysis revealed that both TNM stages and XIST expression could cause differences in overall survival time; furthermore, multivariate analysis revealed that the differences in overall survival time caused by XIST expression were significantly significant (Table 2). The survival time of the patients with thyroid cancer possessing a lower XIST expression was longer (Fig. 1e). To validate the sensitivity and specificity of XIST expression as a biomarker for thyroid cancer, receiver operating characteristic (ROC) curve was employed. The area under the curve (AUC) was 0.7360 (*P* < 0.0001), suggesting the potential of XIST expression as a novel biomarker for thyroid cancer and might help with diagnosis and monitor therapeutic efficacy.

**Table 1:** Correlation of the expression of XIST with clinicopathologic features

Clinic-pathological parameters		XIST expression		*p*-value
		High	Low	
Gender	female	26	28	0.501
	male	13	10	
Age (years)	< 50	21	21	0.901
	≥ 50	18	17	
Tumor size(cm^3^)	< 3	12	21	0.030
	≥ 3	27	17	
N-stage	N0	9	24	< 0.001
	N1	30	14	
TNM	I + II	14	27	0.002
	III + IV	25	11	
Histopathological type	Follicular cancer	12	14	0.807
	Medullary thyroid carcinoma	3	2	
	Papillary cancer	24	22

**Table 2:** Univariate and multivariate analysis for factors related to disease free survival using the COX proportional hazard model

Variable	Univariate analysis			Multivariate analysis		
	*p*	HR	95% CI	*p*	HR	95% CI
Gender						
female vs male	0.155	2.945	0.664–13.052	0.155	2.945	0.664–13.052
Age (years)						
< 50 vs ≥50	0.545	0.731	0.265–2.017			
Tumor size(cm^3)^						
< 3 vs ≥3	0.311	0.573	0.195–1.682			
N-stage						
N0 vs N1	0.294	0.562	0.191–1.649			
TNM						
I + II vs III + IV	0.009	0.185	0.052–0.659	0.138	0.352	0.088–1.401
Histopathological type	0.822					
Follicular cancer vs Papillary cancer	0.535	1.398	0.485–4.030			
Medullary thyroid carcinoma vs Papillary cancer	0.821	1.271	0.159–10.172			
XIST						
4.976 ± 1.918,*n* = 77	< 0.001	1.749	1.288–2.373	0.011	1.545	1.107–2.157

### XIST knockdown inhibits the cell and tumor growth of thyroid cancer

**Fig. 2:**
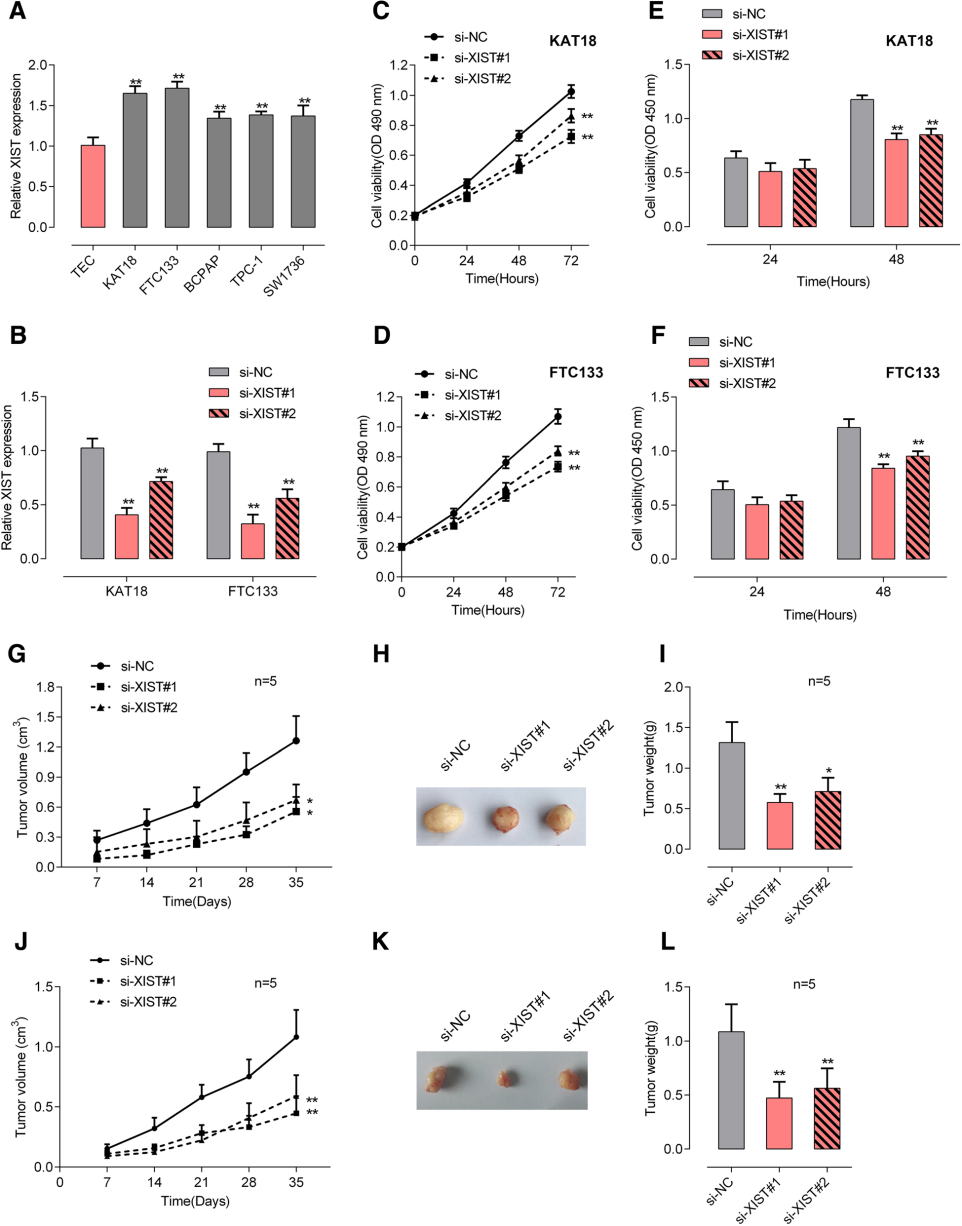
XIST knockdown inhibits the cell and tumor growth of thyroid cancer (**a**) XIST expression in five thyroid cancer cell lines (FTC133, BCPAP, TPC-1, SW1736 and KAT18) and a normal human thyroid epithelium cell line (TEC) was examined using real-time PCR assays. **b** XIST knockdown was achieved by transfection of si-XIST#1 or si-XIST#2, as confirmed using real-time PCR assays. **c**-**d** The cell viability was determined using MTT assays. **e**-**f** The DNA synthesis ability was determined using BrdU assays. **g**-**i** The tumor volume and tumor weight derived from si-XIST#1 or si-XIST#2-transfected KAT18 cells were examined. **j**-**l** The tumor volume and tumor weight derived from si-XIST#1 or si-XIST#2-transfected TPC-1 cells were examined. The data are presented as mean ± SD of three independent experiments. *P < 0.05, **P < 0.01

To further investigate the detailed role and molecular mechanism of XIST in thyroid cancer, five thyroid cancer cell lines, as well as a normal human thyroid epithelium cell line were subjected to real-time PCR assays for XIST expression. Consistent with that in tissue samples, XIST expression was significantly increased in thyroid cancer cell lines, more up-regulated in KAT18 and FTC113 cells, compared to TEC (Fig. 2a). To evaluate the effect of XIST, si-XIST#1 or si-XIST#2 was transfected into KAT18 and FTC113 cell lines to conduct XIST expression, as confirmed by real-time PCR assays (Fig. 2b). Next, MTT and BrdU assays were used to examine the cell growth of transfected KAT18 and FTC113 cells. XIST knockdown significantly suppressed the cell proliferation of KAT18 and FTC113 cells (Fig. 2c-f). Moreover, xenograft tumor models were also constructed in mice with KAT18 and TPC-1 cell lines. We monitored the tumor volume and weight in response to XIST knockdown. As shown in Fig. 2g-l, the volume and weight of tumors derived from both cell lines could be significantly reduced by XIST knockdown. The above indexes were more down-regulated by si-XIST#2, thus si-XIST#2 was chosen as siRNA for XIST for further experiments.

### Screening and verification of candidate XIST downstream target miRNAs

**Fig. 3:**
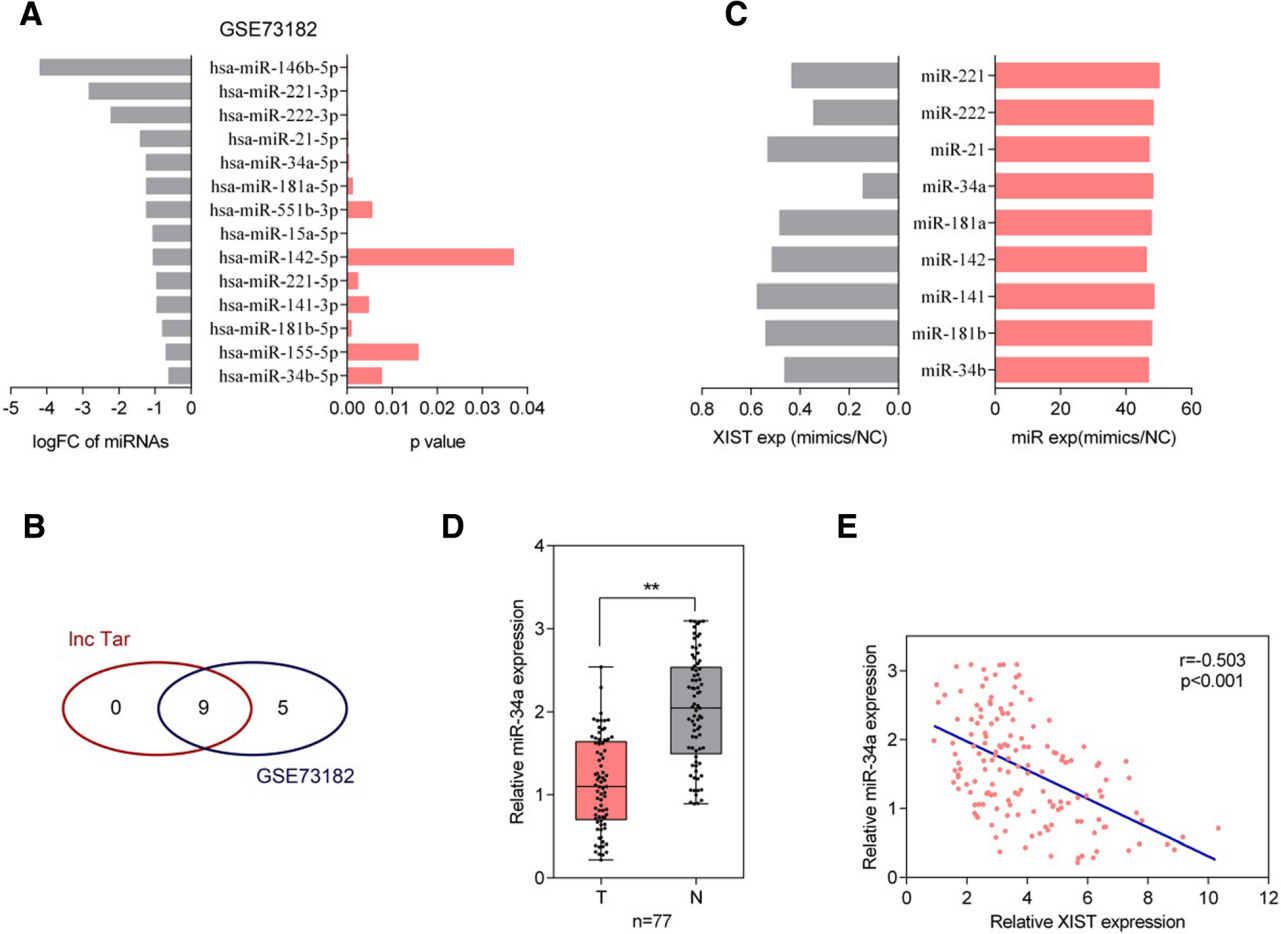
Screening and verification of candidate XIST downstream target miRNAs (**a**) The fold-change of down-regulated miRNAs in thyroid cancer compared to non-tumor tissue samples, showing as log_FC_ and *P* values. **b** Candidate downstream miRNAs of XIST predicted by lnc Tar and the most down-regulated miRNAs reported by GSE73182. (**c**) The expression of the above candidate miRNAs was achieved by transfection of miRNA mimics, as confirmed using real-time PCR assays; XIST expression was then determined using real-time PCR assays. **d** miR-34a expression in 77 paired thyroid cancer and non-tumor tissue samples was examined using real-time PCR assays. The data are presented as mean ± SD of three independent experiments. **P < 0.01. **e** The correlation between miR-34a expression and XIST expression was analyzed using Spearman’s rank correlation analysis

LncRNAs commonly exert their effects through interacting with downstream target miRNAs [27]. To investigate whether XIST modulates the cell proliferation and tumor growth of thyroid cancer through interacting with miRNAs, we searched online database Gene Expression Omnibus (GEO) for dysregulated miRNAs. We selected 14 down-regulated miRNAs (log (FC) < − 0.5 and *P* < 0.05) based on microarray data (GSE73182) [28] (Fig. 3a). Next, lnc Tar online tool (http://www.cuilab.cn/lnctar) [26] was used to identify candidate target miRNAs of XIST. Of the above 14 miRNAs, 9 were predicted to be possible downstream targets of XIST (Fig. 3b). Next, the mimics of the above candidate miRNAs were transfected into FTC33 cells to achieve miRNA expression, as confirmed by real-time PCR assays (Fig. 3c). Next, XIST expression in the above transfected cells was detected by real-time PCR assays. XIST expression was obviously down-regulated by the above miRNAs, more suppressed by miR-34a (Fig. 3c). Thus, miR-34a was chosen as further object. Next, miR-34a expression in 77 paired thyroid cancer and adjacent non-tumor tissue samples was examined using real-time PCR assays; consistent with microarray results, miR-34a expression was significantly reduced in thyroid cancer tissue samples (Fig. 3d). Moreover, miR-34a was negatively correlated with XIST, indicating that they might negatively regulate each other.

### XIST targets miR-34a to negatively interact with miR-34a

**Fig. 4:**
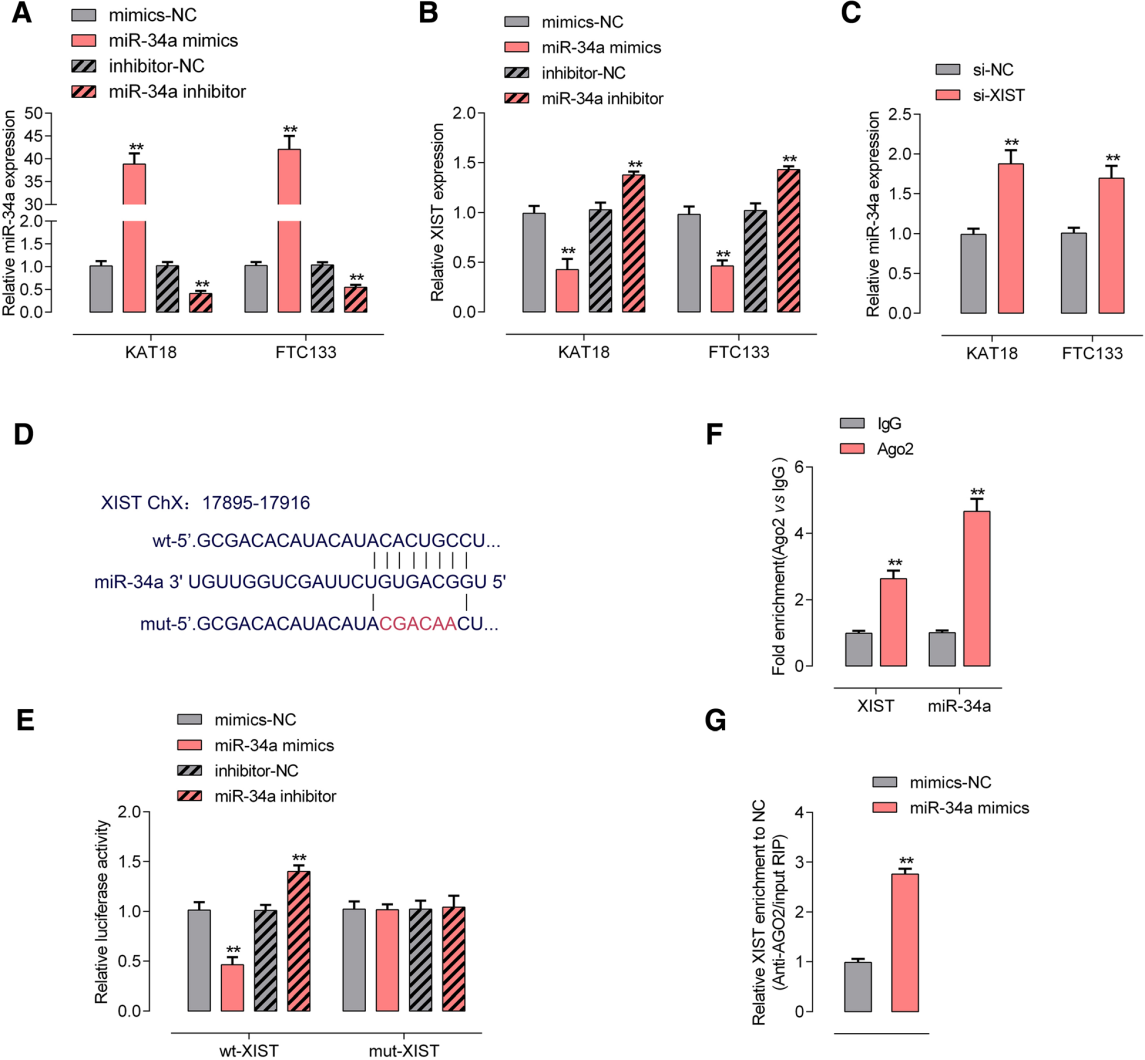
XIST targets miR-34a to negatively interact with miR-34a (**a**) KAT18 and FTC133 cells were transfected with miR-34a mimics or miR-34a inhibitor to achieve miR-34a expression, as confirmed using real-time PCR assays. **b** XIST expression was determined using real-time PCR assays. **c** miR-34a expression in response to XIST knockdown was determined using real-time PCR assays. **d**-**e** Luciferase reporter gene assays were performed to confirm the direct targeting of XIST to miR-34a. **f** Association of miR-34a and XIST with AGO2 in HEK293 cells. Detection of AGO2 and IgG using Immunoblotting assays. **g** RIP assay in HEK293 cells transfected with control miRNA (miR-NC) or miR-34a followed by real-time PCR to detect XIST associated with AGO2. The data are presented as mean ± SD of three independent experiments. **P < 0.01

KAT18 and FTC33 cells were subjected to miR-34a mimics or miR-34a inhibitor transfection to achieve miR-34a expression, as confirmed by real-time PCR assays (Fig. 4a); XIST expression in these cells was examined. As hypothesized, XIST expression was remarkably increased by miR-34a inhibition whereas suppressed by ectopic miR-34a expression (Fig. 4b). Moreover, si-XIST-induced XIST knockdown also significantly increased miR-34a expression (Fig. 4c), indicating that XIST and miR-34a could indeed negatively regulate each other.

**Fig. 5:**
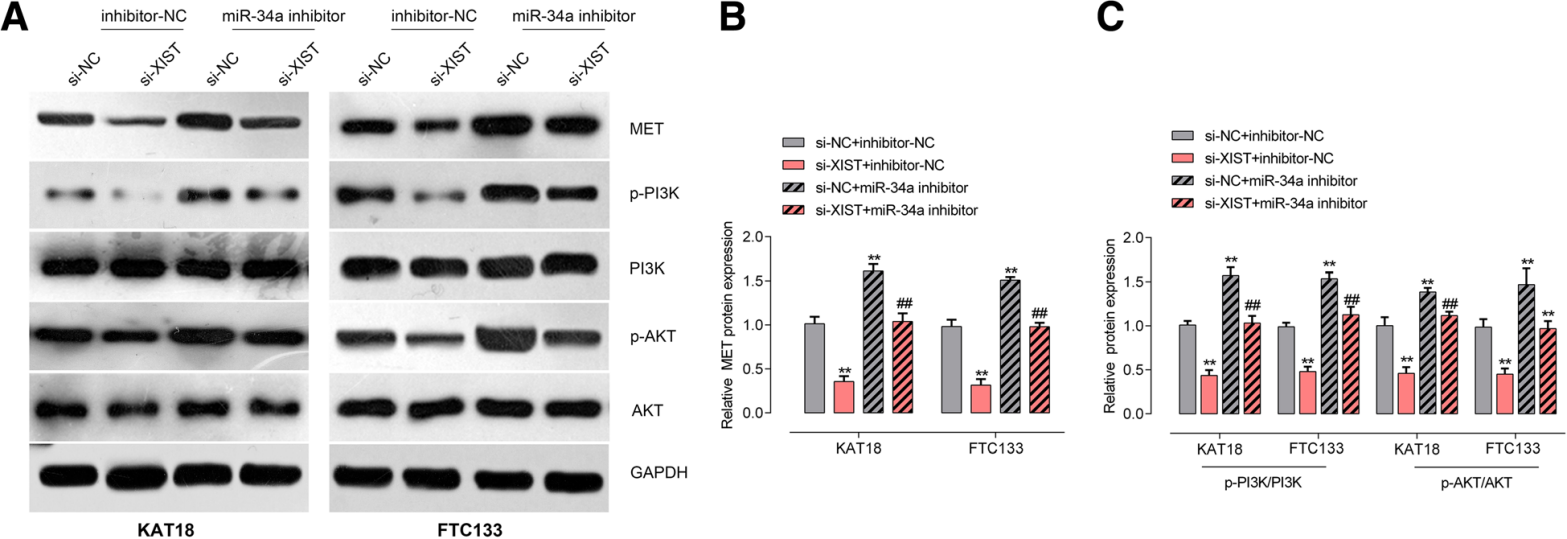
XIST modulates miR-34a downstream MET-PI3K-AKT signaling via miR-34a (**a**-**c**) KAT18 and FTC113 cells were co-transfected with si-XIST and miR-34a inhibitor; the protein levels of MET, p-PI3K, PI3K, p-AKT and AKT were examined using Immunoblotting assays. The data are presented as mean ± SD of three independent experiments. **P < 0.01, compared to control group; ##*P* < 0.01, compared to si-XIST group

To confirm the interaction between XIST and miR-34a, luciferase and RIP assays were performed. Wild-type and mutant-type XIST reporter gene vectors were constructed and named wt-XIST and mut-XIST; mut-XIST vector contained a 6 bp mutation at the predicted miR-34a binding site (Fig. 4d). HEK293 cells were then co-transfected with the above vectors and miR-34a mimics or miR-34a inhibitor; and then the luciferase activity was examined. As shown in Fig. 4e, the luciferase activity of wild-type luciferase reporter gene vector could be dramatically suppressed by miR-34a mimics whereas amplified by miR-34a inhibitor; after mutating the putative miR-34a binding site predicted by online tool, the alternation of luciferase activity caused by miR-34a overexpression or miR-34a inhibition was eliminated. Furthermore, RIP assay showed that XIST and miR-34a were associated with the AGO2 in HEK293 cells. XIST and miR-34a levels were dramatically higher in the RNA derived from precipitated AGO2 protein than those in IgG (Fig. 4f). We also performed RIP assay in HEK293 cells transfected with control miRNA (miR-NC) or miR-34a followed by real-time PCR to detect XIST associated with AGO2; the results shown in Fig. 5g confirmed the interaction between XIST and miR-34a.

### XIST modulates miR-34a downstream MET-PI3K-AKT signaling via miR-34a

Previously, miR-34a has been regarded as a tumor suppressor through targeting MET [29–32], a RTK (receptor tyrosine kinase) playing a key role in promoting cell proliferation via transducing extracellular stimuli to intracellular signalling circuits [33, 34], through PI3K/AKT signaling [31]. Here, we investigated whether MET and PI3K/AKT signaling could be modulated by XIST/miR-34a axis. KAT18 and FTC33 cells were co-transfected with miR-34a inhibitor and si-XIST; next, the protein levels of MET, p-PI3K, PI3K, p-AKT and AKT were examined using Immunoblotting assays. As shown in Fig. 5a and b, MET protein levels were significantly decreased by XIST knockdown whereas increased by miR-34a inhibition; the MET protein levels reduced by XIST knockdown could be significantly rescued by miR-34a inhibition (Fig. 5a and b). Furthermore, the ratio of p-PI3K/PI3K and p-AKT/AKT in these two cell lines could be also reduced by XIST knockdown and increased by miR-34a inhibition; the effect of XIST knockdown on PI3K and AKT phosphorylation was partially attenuated by miR-34a inhibition (Fig. 5a and c), indicating that XIST knockdown could decrease MET protein and the phosphorylation of PI3K and AKT, while miR-34a inhibition could partially reverse the effect of XIST knockdown.

### MET mRNA expression and protein levels in cancer and non-tumor tissue samples and its correlation with XIST and miR-34a

**Fig. 6:**
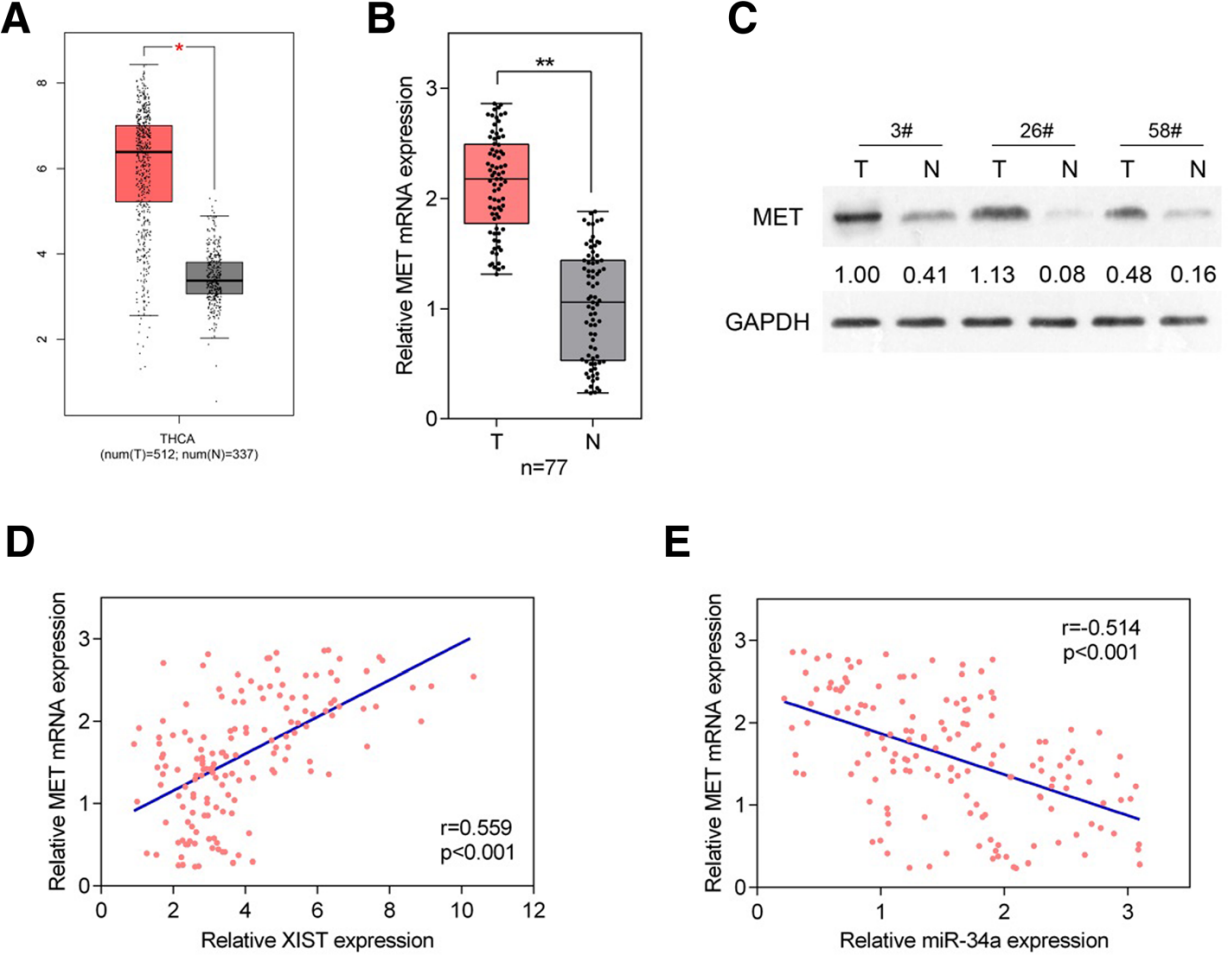
MET expression in tissue samples and its correlation with XIST and miR-34a (**a**) MET mRNA expression in 512 thyroid cancer and 337 non-tumor tissues based on TCGA-THCA database. **b** MET mRNA expression in 77 paired thyroid cancer and non-tumor tissue samples was determined using real-time PCR assays. The data are presented as mean ± SD of three independent experiments. *P < 0.05, **P < 0.01. **c** The protein levels of MET in three paired randomly selected thyroid cancer and non-tumor tissues were determined using Immunoblotting assays. **d**-**e** The correlation between XIST and MET, between miR-34a and MET was analyzed using Spearman’s rank correlation analysis

In order to further confirm the above findings, we analyzed the expression of MET mRNA based on TCGA-THCA database containing 512 thyroid cancer and 337 non-tumor tissues; as shown in Fig. 6a, MET mRNA expression could be remarkably promoted in thyroid cancer tissue samples, compared to that in non-tumor tissue samples. In 77 paired thyroid cancer and non-tumor tissues, MET mRNA expression was also remarkably up-regulated, consistent database results (Fig. 6b). In three paired randomly-selected tissue samples, MET protein levels were increased in cancer tissues, compared to those in non-tumor tissue samples (Fig. 6C). Furthermore, the Spearman’s rank correlation analysis revealed that MET mRNA expression was positively correlated with XIST whereas negatively correlated with miR-34a (Fig. 6d-e). These data further indicate that XIST targets miR-34a to modulate the cell proliferation and tumor growth through miR-34a downstream MET and PI3K/AKT signaling.

## Discussion

**Fig. 7:**
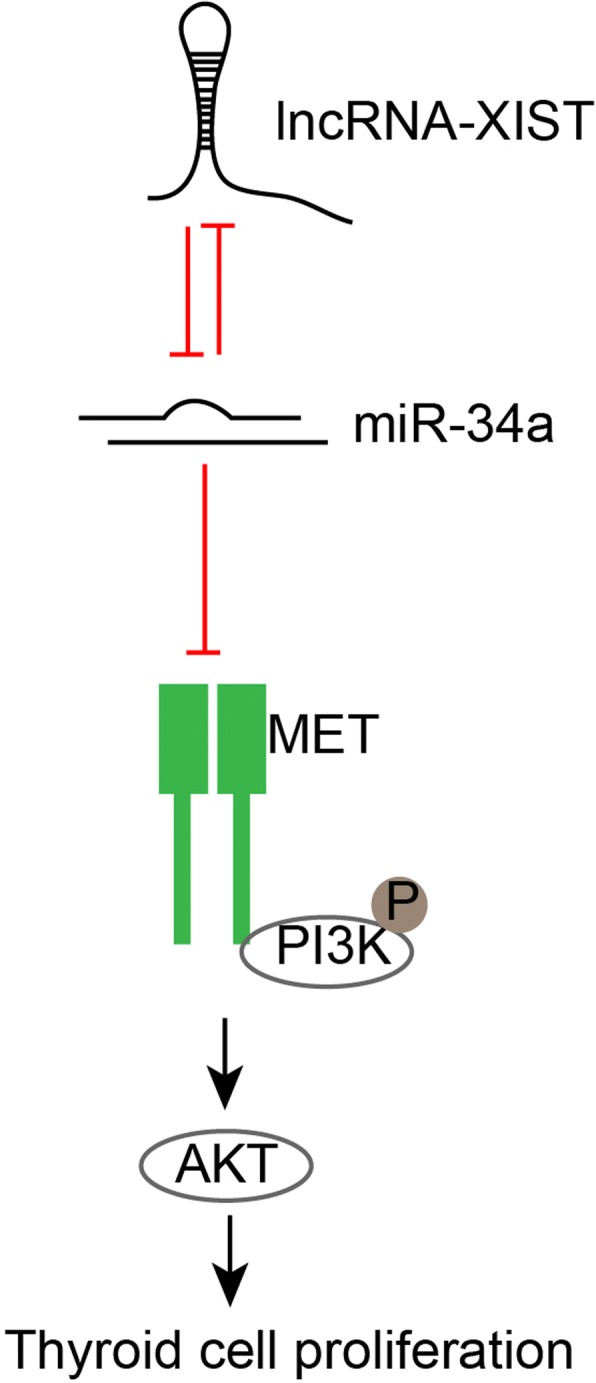
Mechanism map

Here, we demonstrate that XIST negatively interacts with miR-34a to modulate the cell proliferation and tumor growth of thyroid cancer through miR-34a downstream MET-PI3K/AKT signaling (Fig. 7).

Over the past decades, aberrant expression of lncRNA has been widely observed and has been reported to correlate with tumorigenesis. Lan et al. [35] demonstrated a genome-wide analysis (GWAS) on the expression profile of lncRNA in papillary thyroid cancer identifying a large amount of up- and down-regulated lncRNAs in cancer samples. Another microarray analysis based on human lncRNA identified the dysregulated expression of 675 lncRNAs in three pairs of thyroid cancer compared with adjacent normal thyroid tissue samples [36]. Herein, we searched TCGA-THCA for dysregulated lncRNAs, among which XIST is considered to be significantly increased in thyroid cancer compared to normal non-tumor tissues. Moreover, the oncogenic role of XIST in cancers has been well-established. Herein, we also observed an up-regulated expression of XIST in 77 thyroid cancer tissues. More importantly, higher XIST expression was correlated with poorer prognosis in patients with thyroid cancer, indicating the potential role of XIST in thyroid cancer pathopoiesis.

LncRNAs have captured increasing attention due to their crucial roles in biological processes, both normal physiological processes and pathogenic processes [37–39]. Reportedly, XIST participates in the proliferation, apoptosis, invasion, migration and resistance to chemo- and radio-therapies of cancer cells. In gastric cancer, XIST affects the cell proliferation, migration and invasion, as well as tumor growth through sponging miR-101 [19]. In bladder cancer, XIST modulates the proliferation, invasion and migration of cancer cells via interacting with miR-124, [20]. In the present study, we revealed that XIST knockdown dramatically suppressed the cell proliferation of thyroid cancer cells in vivo, and the tumor growth in vitro, which is consistent with previous studies in other cancers.

It has been widely regarded that lncRNA-miRNA-mRNA interactions play a crucial role in tumorigenesis [40–42]. As we mentioned, XIST exerts its biological effects in cancers commonly through interacting with different miRNAs [18–23, 25]. To further understand the molecular mechanism of XIST in thyroid cancer, we searched GEO database for dysregulated miRNAs in thyroid cancer tissues. Minna et al. reported a series of 18 miRNAs remarkably dysregulated (|fold-change| ≥ 1.5; FDR < 0.05) in thyroid cancer tissues compared to non-tumor thyroid tissues [28]; of the reported miRNAs, miR-34a inhibited XIST expression in thyroid cancer cells the most significantly in the present study. In addition to the negative regulation between XIST and miR-34a, we also confirmed that miR-34a was a direct downstream target of XIST, as predicted by lnc Tar [26]. Regarding the downstream transcripts of miR-34a, we investigated whether MET, which is considered as a direct downstream transcript of miR-34a [29–32], as well as PI3K/AKT signaling could be modulated by XIST/miR-34a axis. Consistent with the above previous studies, XIST knockdown could suppress the protein levels of MET and the phosphorylation of PI3K and AKT, whereas miR-34a inhibition could partially attenuate the effect of XIST knockdown, indicating that XIST could modulate MET-PI3K/AKT signaling through miR-34a. In thyroid cancer, XIST may serve a ceRNA for miR-34a to compete with MET for miR-34a binding, thus affecting thyroid cancer cell proliferation and tumor growth.

To further confirm the above findings, we monitored MET mRNA expression and protein levels in thyroid cancer and non-tumor tissues. Contrary to miR-34a, MET mRNA expression and protein level were significantly increased in thyroid cancer tissue samples; moreover, MET expression was positively correlated with XIST, whereas negatively correlated with miR-34a expression.

## Conclusion

XIST serves as a ceRNA for miR-34a through sponging miR-34a, competing with MET for miR-34a binding, and finally modulating thyroid cancer cell proliferation and tumor growth. More importantly, the cell lines we chose here were derived from the major pathological type of thyroid cancer, thus, these findings may provide a solid experimental basis for the in-depth understanding of thyroid cancer pathology.

## Additional file

Additional file 1: **Table S1.** Top 10 significantly expressed lncRNAs in thyroid cancer. **Table S2.** Sequences. (DOCX 21 kb)

## Abbreviations

AKT: α-serine/threonine-protein kinase
ATC: Anaplastic thyroid cancer
FTC: Follicular thyroid cancer
GEO: Gene Expression Omnibus
GWAS: Genome-wide analysis
lncRNA: Long non-coding RNA
MET: Hepatocyte growth factor receptor
PDTC: Partially differentiated thyroid cancer
PI3K: Phosphoinositide 3-kinase
PTC: Papillary thyroid cancer
RIP: RNA Immunoprecipitation
XIST: X-inactive specific transcript
