# Supplementary material for: LncRNA XIST/miR-34a axis modulates the cell proliferation and tumor growth of thyroid cancer through MET-PI3K-AKT signaling
Source: J Exp Clin Cancer Res. 2018 Nov 21;37:279. doi: 10.1186/s13046-018-0950-9 (PMC6249781; doi:10.1186/s13046-018-0950-9)
Supplement: Supplementary file 1 — Table S1. Top 10 significantly expressed lncRNAs in thyroid cancer. Table S2. Sequences. (DOCX 21 kb) [file 13046_2018_950_MOESM1_ESM.docx]

Table S1 Top 10 significantly expressed lncRNAs in thyroid cancer

| **Gene Symbol** | **Gene ID** | **Median (Tumor)** | **Median (Normal)** | **Log2(Fold Change)** | **adjp** |
| --- | --- | --- | --- | --- | --- |
| RP11-40C6.2 | ENSG00000219928.2 | 993.515 | 1.89 | 8.427 | 6.74E-164 |
| [DCSTAMP](http://gepia.cancer-pku.cn/detail.php?gene=DCSTAMP) | ENSG00000164935.6 | 94.382 | 0.12 | 6.412 | 1.06E-83 |
| [ZCCHC12](http://gepia.cancer-pku.cn/detail.php?gene=ZCCHC12) | ENSG00000174460.3 | 148.446 | 0.99 | 6.231 | 6.69E-121 |
| [SERPINA1](http://gepia.cancer-pku.cn/detail.php?gene=SERPINA1) | ENSG00000197249.12 | 1367.096 | 24.21 | 5.762 | 4.70E-117 |
| [XIST](http://gepia.cancer-pku.cn/detail.php?gene=XIST) | **ENSG00000229807.9** | **108.146** | **1.16** | **5.659** | **7.82E-29** |
| [FN1](http://gepia.cancer-pku.cn/detail.php?gene=FN1) | ENSG00000115414.18 | 3944.699 | 80.092 | 5.605 | 5.44E-92 |
| [CITED1](http://gepia.cancer-pku.cn/detail.php?gene=CITED1) | ENSG00000125931.10 | 278.222 | 5.61 | 5.401 | 1.97E-125 |
| [KLK10](http://gepia.cancer-pku.cn/detail.php?gene=KLK10) | ENSG00000129451.11 | 56.33 | 0.53 | 5.228 | 4.78E-94 |
| [RP5-940J5.9](http://gepia.cancer-pku.cn/detail.php?gene=RP5-940J5.9) | ENSG00000269968.1 | 35.554 | 0 | 5.192 | 2.61E-53 |
| TACSTD2 | ENSG00000184292.6 | 185.772 | 4.37 | 5.12 | 2.26E-76 |

Table S2 Sequences

| Name | Sequences |
| --- | --- |
| hsa-miR-34b-5p-RT | 5’-GTCGTATCCAGTGCGTGTCGTGGAGTCGGCAATTGCACTGGATACGACCAATCA-3’ |
| hsa-miR-34b-5p-F | 5’-GCCTAGGCAGTGTCATTAGC-3’ |
| hsa-miR-181b-5p-RT | 5’-GTCGTATCCAGTGCGTGTCGTGGAGTCGGCAATTGCACTGGATACGACACCCAC-3’ |
| hsa-miR-181b-5p-F | 5’-GCAACATTCATTGCTGTCG-3’ |
| hsa-miR-141-5p-RT | 5’-GTCGTATCCAGTGCGTGTCGTGGAGTCGGCAATTGCACTGGATACGACTCCAAC-3’ |
| hsa-miR-141-5p-F | 5’-GCCCATCTTCCAGTACAGT-3’ |
| hsa-miR-142-5p-RT | 5’-GTCGTATCCAGTGCGTGTCGTGGAGTCGGCAATTGCACTGGATACGACAGTAGT-3’ |
| hsa-miR-142-5p-F | 5’-GCCGCATAAAGTAGAAAGC-3’ |
| hsa-miR-181a-5p-RT | 5’-GTCGTATCCAGTGCGTGTCGTGGAGTCGGCAATTGCACTGGATACGACACTCAC-3’ |
| hsa-miR-181a-5p-F | 5’-GAACATTCAACGCTGTCG-3’ |
| hsa-miR-21-5p-RT | 5’-GTCGTATCCAGTGCGTGTCGTGGAGTCGGCAATTGCACTGGATACGACTCAACA-3’ |
| hsa-miR-21-5p-F | 5’-GCCGTAGCTTATCAGACTGA-3’ |
| hsa-miR-221-5p-RT | 5’-GTCGTATCCAGTGCGTGTCGTGGAGTCGGCAATTGCACTGGATACGACAAATCT-3’ |
| hsa-miR-221-5p-F | 5’-GCACCTGGCATACAATGT-3’ |
| hsa-miR-34a-5p-RT | 5’-GTCGTATCCAGTGCGTGTCGTGGAGTCGGCAATTGCACTGGATACGACACAACC-3’ |
| hsa-miR-34a-5p-F | 5’-GCTGGCAGTGTCTTAGCT-3’ |
| hsa-miR-R | 5’-CAGTGCGTGTCGTGGA-3’ |
| U6-F | 5’-CTCGCTTCGGCAGCACA-3’ |
| U6-R | 5’-AACGCTTCACGAATTTGCGT-3’ |
| XIST-158F | 5’-TTGGGGAACCACCTACACTTGAG-3’ |
| XIST-158R | 5’-CCATTTTGCTATGCGTTATCTGA-3’ |
| MET-198F | 5’AGCAATGGGGAGTGTAAAGAGG-3’ |
| MET-198R | 5’CCCAGTCTTGTACTCAGCAAC-3’ |
| GAPDH-F | 5’-ACAGCCTCAAGATCATCAGC-3’ |
| GAPDH-R | 5’-GGTCATGAGTCCTTCCACGAT-3’ |
| miR-34a inhibitor | 5’-ACAACCAGCUAAGACACUGCCA-3’ |
| inhibitor NC | 5'-CAGUACUUUUGUGUAGUACAA-3' |
| Si-XIST-1F | UUAAGUAGUAGGUACUUCCAG |
| Si-XIST-1R | GGAAGUACCUACUACUUAAGA |
| Si-XIST-2F | UUAAUGUCCAAUAAUGUCCAA |
| Si-XIST-2R | GGACAUUAUUGGACAUUAAUG |
| SiRNA-NC-F | TTCTCCGAACGTGTCACGT |
| SiRNA-NC-R | ACGTGACACGTTCGGAGAA |
| XIST-WT-F | 5’-CCGCTCGAGAACCACCTACACTTGAGCC-3’ |
| XIST-WT-R | 5’-ATAAGAATGCGGCCGCTTTCCTTCACTCTTCCTCC-3’ |
| XIST-MUT-F | 5’-ATGCTGACACATACATACGACAACTAAATAGATCTCTTTC-3’ |
| XIST-MUT-R | 5’-GAAAGAGATCTATTTAGTTGTCGTATGTATGTGTCAGCAT-3’ |
| psiCHECK2 | >NR_001564.2 Homo sapiens X inactive specific transcript (XIST), long non-coding RNA CAAATATATTCGGGTGCCAATAGGTACTTGGTATAAGGTTTTTGGCCCCAGAGACATGGGAAAAAAATGC ATGCCTTCCCAGAGAATGCCTAATACTTTCCTTTTGGCTTGTTTTCTTGTTAGGGGCATGGCTTAGTCCC TAAATAACATTGTGTGGTTTAATTCCTACTCCGTATCTCTTCTACCACTCTGGCCACTACGATAAGCAGG TAGCTGGGTTTTGTAGTGAGCTTGCTCCTTAAGTTACAGGAACTCTCCTTATAATAGACACTTCATTTTC CTAGTCCATCCCTCATGAAAAATGACTGACCACTGCTGGGCAGCAGGAGGGATGATGACCAACTAATTCC CAAACCCCAGTCTCATTGGTACCAGCCTTGGGGAACCACCTACACTTGAGCCACAATTGGTTTTGAAGTG CATTTACAAGGTTTGTCTATTTTCAGTTCTTTACTTTTTACATGCTGACACATACATACACTGCCTAAAT AGATCTCTTTCAGAAACAATCCTCAGATAACGCATAGCAAAATGGAGATGGAGACATGATTTCTCATGCA ACAGCTTCTCTAATTATACCTTAGAAATGTTCTCCTTTTTATCATCAAATCTGCTCAAGAAGGGCTTTTT ATAGTAGAATAATATCAGTGGATGAAAACAGCTTAACATTTTACCATGCTTAAGTTTTAAGAATAAAATA AAAATTGGAAATAATTGGCCAAAATTGAAAGGAAAAATTTTTTTAAAATTTCTCTAAATGTAGGCCTGGC TGGGCTTTGACCTTTTCCGTTTTTAAATCACTCACAGAGGGTGGGACAGGAGGAAGAGTGAAGGAAAAGG TCAAACCTGTTTTAAGGGCAACCTGCCTTTGTTCTGAATTGGTCTTAAGAACATTACCAGCTCCAGGTTT AAATTGTTCAGTTTCATGCAGTTCCAATAGCTGATCATTGTTGAGATGAGGACAAAATCCTTTGTCCTCA CTAGTTTGCTTTACATTTTTGAAAAGTATTATTTTTGTCCAAGTGCTTATCAACTAAACCTTGTGTTAGG |
